# Selenium Reduces Cadmium-Induced Cardiotoxicity by Modulating Oxidative Stress and the ROS/PARP-1/TRPM2 Signalling Pathway in Rats

**DOI:** 10.3390/toxics13080611

**Published:** 2025-07-22

**Authors:** Yener Yazğan, Ömer Faruk Keleş, Mehmet Hafit Bayir, Hacı Ahmet Çiçek, Adem Ahlatcı, Kenan Yıldızhan

**Affiliations:** 1 Department of Biophysics, Faculty of Medicine, Kastamonu University, Kastamonu 37100, Türkiye; yener8275@hotmail.com; 2 Department of Pathology, Faculty of Veterinary Medicine, Van Yuzuncu Yil University, Van 65080, Türkiye; haciahmet99@gmail.com; 3 Department of Histology, Faculty of Medicine, Van Yuzuncu Yil University, Van 65080, Türkiye; mehmethafitbayir@yyu.edu.tr; 4 Vocational School of Health Services, Van Yuzuncu Yil University, Van 65080, Türkiye; ademahlatci@yyu.edu.tr; 5 Department of Biophysics, Faculty of Medicine, Van Yuzuncu Yil University, Van 65080, Türkiye; kenanyldzhan@gmail.com

**Keywords:** cadmium, cardiotoxic, oxidative stress, selenium, TRPM2 channel, histopathology

## Abstract

Cadmium (CAD) is a prevalent environmental contaminant that poses serious cardiotoxic risks. The heart, kidney, liver, and brain are just a few of the essential organs that can sustain serious harm from CAD, a very poisonous heavy metal. The cardiotoxic mechanism of CAD is linked to oxidative damage and inflammation. A trace element with anti-inflammatory, anti-apoptotic, and antioxidant qualities, selenium (SEL) can be taken as a dietary supplement. The biotoxicity of heavy metal CAD is significantly inhibited by SEL, a mineral that is vital to human and animal nutrition. Through ROS-induced PARP-1/ADPR/TRPM2 pathways, this study seeks to assess the preventive benefits of selenium against cardiovascular damage caused by CAD. The SEL showed encouraging results in reducing inflammatory and oxidative reactions. Rats were given 0.5 mg/kg SEL and 3 mg/kg 2-Aminoethyl diphenylborinate (2-APB) intraperitoneally for five days, in addition to 25 mg/kg CAD given via gavage. Histopathological examination findings revealed that the morphologic changes in the hearts of the CAD group rats were characterised by marked necrosis and the degeneration of myocytes and congestion of vessels. Compared to the rats in the CAD group, the hearts of the SEL, 2-APB and SEL+2-APB groups showed fewer morphological alterations. Moreover, in rats given CAD, there was an increase in cardiac malondialdehyde (MDA), total oxidant (TOS), reactive oxygen species (ROS), caspase (Casp-3-9), and TNF-α, whereas glutathione (GSH), glutathione peroxidase (GSH-Px), superoxide dismutase (SOD) and total antioxidant (TAS) decreased. SEL improved antioxidants, avoided tissue damage, and reduced cardiac MDA, TOS, and ROS. In rats given CAD, SEL decreased cardiac PARP-1, TRPM2, TNF-α, and caspase. In summary, by reducing oxidative stress and cardiac damage and modifying the ROS/PARP-1/TRPM2 pathway, SEL protected against CAD cardiotoxicity.

## 1. Introduction

Cadmium (CAD) is a heavy metal that poses major risks to human health and has harmful effects. Human exposure has dramatically risen and CAD is now acknowledged as an industrial and environmental contaminant [1]. It is easily transported from the soil to plants and builds up in significant quantities in the food chain, much like other dangerous heavy metals [2]. One of the most prevalent harmful heavy metals, CAD poses serious cardiotoxic risks, particularly when exposed in excess from cigarette smoking or contaminated food and water. CAD can build up in our bodies for a very long time. The production of oxidative damage and disturbance of the oxidant/antioxidant balance have been associated with the harmful impact of CAD [1,2].

Exposure to metals such as arsenic, cadmium, chromium, and lead has been shown to cause heart dysfunction and hypertension in rodent models [3]. Even at modest dosages, CAD exposure harms the cardiovascular system and other organ systems. Atherosclerosis, myocardial infarction, and cardiac hypertrophy are among the cardiovascular conditions that are associated with CAD. Although several theories have been proposed to explain CAD-induced cardiac dysfunction, the precise mechanism or processes are unclear. According to reports, the harmful effects of CAD change the quantity of nitric oxide in endothelial cells, which may cause the increased levels of reactive oxygen species (ROS) generation and lipid peroxidation [4,5]. The ability of CAD to impair a variety of cellular functions and harm many cellular constituents characterises its toxicity [6,7]. CAD exposure has been connected to a multitude of cardiovascular conditions, including atherosclerosis, stroke, hypertension, and cardiac arrest [8]. The main risk factors for atherosclerosis development include an imbalance in lipid indicators, particularly plasma lipoprotein and cholesterol levels, and disturbance of the antioxidant system [9]. Studies have revealed that CAD builds up in the heart and arteries of both people and experimental animals, despite the fact that the liver and kidneys are the primary locations for CAD deposition [10,11]. Egger et al. demonstrated the accumulation of CAD in cardiac tissue through measurements of CAD concentrations in human cadaver samples [10]. Similarly, Gerzen et al. showed that both lead and CAD enter cardiomyocytes and distribute throughout the cells, impairing their contractile function, as evidenced by the use of Leadmium Green staining [12]. Rats given CAD showed signs of inflammation, and their hearts produced more pro-inflammatory cytokines [4]. CAD caused ROS to be produced, which accumulated in cardiomyocytes in vitro and led to inflammatory signalling activation, dysfunction, and cell death via apoptosis [13].

Many biological systems, including the cardiovascular system, depend on selenium (SEL), a trace element that is vital and necessary for life. SEL deficiency is significantly linked to a number of illnesses that represent a serious risk to human health [14]. Selenoproteins may potentially benefit the cardiovascular system because of their antioxidant properties, which boost antioxidant capacity and lower ROS production [15]. In addition, it has been demonstrated that severe inflammatory disorders, such as systemic inflammatory response syndrome, are linked to low plasma SEL levels. It has been suggested that by lowering inflammation, SEL supplements may enhance myocardial function [14,16]. Low SEL levels disrupt the production of a specific subset of selenoproteins, including glutathione peroxidase, under stress. This disturbance leads to a lack of one or more of these vital proteins in the heart, which may impact the cardiovascular system as a whole [17].

According to reports, SEL supports healthy cardiovascular system function [14]. In cases of SEL deficiency, oxidative stress-related cardiovascular disorders have been shown [18]. By scavenging ROS and nitrogen, SEL supplementation at various dosages and formulations lessens myocardial ischemia/reperfusion damage. Cardiotoxicity from CAD may be exacerbated by SEL or glutathione (GSH) depletion. By strengthening antioxidant defence, SEL treatment reduced CAD-induced cardiotoxicity [19,20]. According to studies, SEL can be a helpful treatment strategy to lessen adverse effects brought on by exposure to hazardous substances or the use of anticancer medications.

Cell calcium signalling is known to be significantly influenced by transient receptor potential (TRP) channels. The cation channels of the TRP family are not selective. Temperature sensing, chemosis, mechanoreceptors, nociception, and photoreceptors are among their vital physiological roles [14]. TRP melastatin 2 (TRPM2) channels are calcium-permeable, non-selective cation channels that have been found in a variety of organs, including the kidney, heart, and most notably the brain and bone marrow [14,21]. Research has demonstrated that oxidative stress (OS) activates TRPM2 channels [22]. By lowering the OS in the environment, SEL has been shown to reduce cell or tissue damage in several investigations on the detrimental consequences of TRPM2 channel activation [23,24].

Studies addressing the function of the TRPM2 channel and the preventive effects of SEL and 2-Aminoethyl diphenylborinate (2-APB) against CAD-induced heart damage were not found in our examination of the literature. Thus, our goal was to look into how the TRPM2 channel contributes to CAD-induced cardiac damage as well as the preventive effects of SEL and 2-APB.

## 2. Materials and Methods

### 2.1. Groups of Experiments

The study used 40 male Albino Wistar rats that were two to three months old and weighed between 200 and 300 g. The Van Yuzuncu Yil University Experimental Animals Local Ethics Committee approved the experiment on 26 September 2024, with approval procedure number 2024/09-02. Five groups were randomly selected from among the eight rats. Rats were housed at 24 °C in plastic cages with 12-h photoperiods of light and dark. During the study, the animals were given unrestricted access to regular feed and drinking water. The details of our previous study are available. Often utilised as pellets, this commercial standard rat feed is meant to provide rats with enough nutrition [14]. The effective dose and duration of CAD (Cadmium chloride, Cat. 655198, Sigma-Aldrich Co., St. Louis, MO, USA) were calculated using the previous CAD’s effective dosage and duration [25]. Taking into consideration the study of Keleş et al., the effective dose and duration of SEL Sodium selenite (Cat; 214485, Sigma-Aldrich Co., St. Louis, MO, USA) were given intraperitoneally at 0.5 mg/kg daily for 5 days [26]. Our results led to modifications and implementation of the adequate dose levels and duration of 2-APB (2-APB, Cat; D9754, Sigma-Aldrich Co., St. Louis, MO, USA) used by Thapak et al. [27]. There were five groups in our study, as may be seen below.

#### Experimental Groups

**CON (Control)**: For five days, the rats in this group were fed regular pellet feed every day.
**CAD:** For five days, the rats in this group received a gavage of 25 mg/kg CAD.
**SEL:** For five days, this group received 25 mg/kg of CAD via gavage, and SEL was given intraperitoneally daily (0.5 mg/kg).
**2-APB:** For five days, this group received 25 mg/kg of CAD via gavage, and 2-APB was injected intraperitoneally daily (3 mg/kg).
**SEL+2-APB**: For five days, this group received 25 mg/kg of CAD via gavage, and both SEL (0.5 mg/kg) and 2-APB (3 mg/kg) were given intraperitoneally daily.

The research was concluded on the fifth day. Rats were fully sedated with xylazine (20 mg/kg) and ketamine (50 mg/kg) at the same centre’s laboratory before tissue samples were collected. A subset of the samples was kept at −80 °C for biochemical analysis. For histological and immunohistochemical analysis, a portion of the hearts was submerged in a 10% formaldehyde solution.

### 2.2. Histopathological Analyses

Following euthanasia, cardiac tissue samples were promptly excised from the rats and fixed in 10% neutral-buffered formalin for at least 24 h to preserve tissue morphology. After fixation, the samples underwent routine histological processing, which included dehydration through a graded ethanol series, clearing in xylene, and infiltration with paraffin wax. The processed tissues were then embedded in paraffin blocks. Serial sections of 4 µm thickness were cut using a rotary microtome and mounted onto glass slides. The sections were deparaffinised in xylene, rehydrated through a descending ethanol series, and stained with hematoxylin and eosin (H&E) using standard protocols to evaluate general histoarchitecture. The stained slides were examined under a light microscope (Nikon 80i -DSRI2, Nikon Imaging Japan Inc., Tokyo, Japan) at various magnifications to assess morphological features.

### 2.3. Immunohistochemical Analyses

Immunohistochemistry was performed using the streptavidin-biotin-peroxidase (avidin–biotin peroxidase complex) method. Briefly, paraffin-embedded tissue sections were deparaffinised in xylene (2 × 5 min) and rehydrated through a graded ethanol series. Endogenous peroxidase activity was blocked by incubating the sections in 3% hydrogen peroxide (H_2_O_2_, *v*/*v*) for 20 min, followed by three 5-min washes in phosphate-buffered saline (PBS). Antigen retrieval was carried out by incubating the slides in citrate buffer (pH 6.0) at 95 °C for 30 min using a water bath, then cooling at room temperature for 20 min. The sections were incubated with blocking serum from the Histostain^®^ Plus Bulk Kit (Zymed Laboratories Inc., South San Francisco, CA, USA) to block nonspecific antibody binding for 15 min. Following blocking, the sections were incubated overnight at 4 °C with the following primary antibodies: TNF-α (Santa Cruz, Santa Cruz, CA, USA, Sc-52746; 1/100 dilution) and Caspase-3 (Abcam, Cambridge, UK, ab 4051; 1/100 dilution). The next day, the slides were washed four times with PBS and incubated with a biotinylated secondary antibody (Histostain^®^ Plus Bulk Kit, Zymed, Thermo Fisher Scientific，Waltham, MA, USA) for 20 min at room temperature. After four additional PBS washes, the sections were incubated with horseradish peroxidase (HRP)-conjugated streptavidin (Histostain^®^ Plus Bulk Kit, Zymed) for 20 min at room temperature. Immunoreactivity was visualized using 3,3′-diaminobenzidine (DAB) as the chromogen for 5–15 min. The slides were then rinsed in distilled water (3 × 5 min), counterstained with Gill’s hematoxylin for 3 min, dehydrated in a graded alcohol series, cleared in xylene, and mounted using Entellan^®^ mounting medium. Immunohistochemical staining intensity was evaluated semi-quantitatively under a light microscope (Olympus BX53, Olympus, Tokyo, Japan) and graded as negative (–), weak (+), moderate (++), or strong (+++), based on visual assessment [28].

### 2.4. Biochemical Evaluation

Following the kit instructions, commercially available ELISA kits were used to quantify the levels of TRPM2 (Bioassay Technology Laboratory, BT LAB, Shanghai, China), PARP-1 (Bioassay Technology Laboratory, BT LAB, China), MDA, GSH, GSH-Px, SOD (Shanghai YL Biotech Co., Ltd., Shanghai, China) and ROS (Sun.Red Biotech Comp. Ltd., Shanghai, China), and Casp-9 (Sun.Red Biotech Comp. Ltd., China) in heart tissues. The amount of protein was calculated by comparing the obtained absorbance values with the standard curve. Our preceding works provide a detailed explanation of the previous methods for protein levels [29].

### 2.5. Antioxidant/Oxidant Levels in Heart Tissues

The supernatant fractions collected from the samples were used to assess the total antioxidant status (Reed Biotech, Wuhan, China) and total oxidant status (Reed Biotech, Wuhan, China) by the test procedures outlined in the appropriate kit protocols. An ELISA microplate reader was used to detect absorbance after each sample was combined with Reagent 1 (TAS at 660 nm; TOS at 530 nm) and then incubated. Using the same wavelengths, a second absorbance measurement was performed following the addition of Reagent 2. A Trolox-equivalent standard curve (1 mmol/L) provided by the kit was used for TAS measurement. Hydrogen peroxide served as the reference standard for TOS analysis, and the results were expressed as micromolar H_2_O_2_ equivalents per litre.

### 2.6. Statistical Analysis

All the data were expressed using the mean ± standard error of the mean (SEM). The Levene test was utilised to confirm that the variances of the independent groups were homogenous, and the Shapiro–Wilk test was utilised to evaluate the normality of the data. After the experiment, every biochemical indicator showed a normal distribution. Since the data were normally distributed, the post hoc (Tukey HSD) test was employed after the one-way ANOVA analysis to determine which groups independently caused the difference. The 5% threshold for statistical significance was used. IBM SPSS 21.0 Statistics for Windows (IBM Corp., Armonk, NY, USA) was used for statistical computations.

## 3. Results

### 3.1. Histopathological Findings

**Figure 1:**
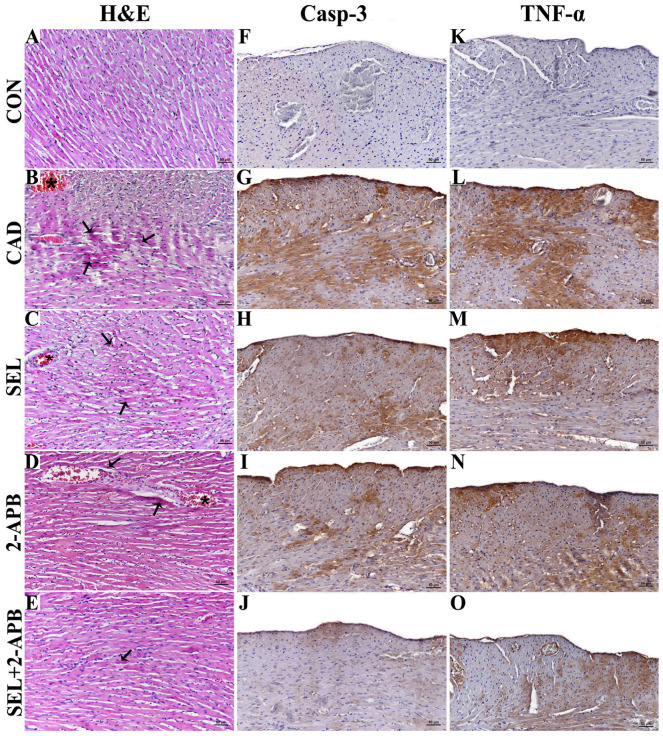
**Histopathological analyses** (**A**–**E**)**;** Hematoxylin and eosin (**H**,**E**)-stained light microscopic pictures of cardiac tissue are used for histopathological examinations. (**A**) (CON): Normal histological appearance of the heart is observed. (**B**) (CAD): Degenerations and necrosis in the myocyte (arrows), and vascular congestion (*). (**C**) (SEL): Necrosis and degeneration in the myocyte (arrows), and vascular congestion (*). (**D**) (2-APB): Necrosis and degeneration in the myocyte (arrow), vascular congestion (*). (**E**) (SEL+2-APB Group): Renal tubular epithelium degenerations and necrosis (arrow). (Bar: 50 µm). **Immune expression of Caspase-3** (**F**–**J**)**;** Effects of cadmium (CAD), selenium (SEL) and 2-Aminoethyl diphenylborinate (2-APB) on the immune expression of Casp-3 in the rat heart tissues: (**F**): CON, (**G**): CAD, (**H**): SEL, (**I**): 2-APB, (**J**): SEL+2-APB. (Bar: 50 µm). **Immune expression of TNF-α** (**K**–**O**)**;** Effects of CAD, SEL and 2-APB on the immune expression of TNF-α in the rat heart tissues: (**K**): CON, (**L**): CAD, (**M**): SEL, (**N**): 2-APB, (**O**): SEL+2-APB. (Bar: 50 µm).

**Figure 2:**
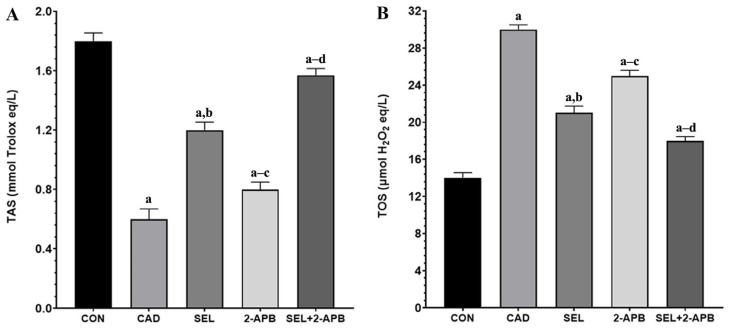
The impact of 2-Aminoethyl diphenylborinate (2-APB) and selenium (SEL) on total antioxidant (TAS) (**A**) and total oxidant (TOS) (**B**) values in cardiac damage caused by cadmium (CAD) (mean ± SD, and *n* = 8). [^a^ *p* < 0.05 comp. CON, ^b^ *p* < 0.05 comp. CAD, ^c^ *p* < 0.05 comp. SEL, ^d^ *p* < 0.05 comp. 2-APB groups, and *p*-values were obtained using a one-way ANOVA test (post hoc Tukey) results].

Normal histological structure of the heart was observed in the tissue sections of the control group (Figure 1A). Morphological changes in the hearts of the CAD group rats included significant necrosis and degeneration in the myocytes, as well as congestion in the vessels (Figure 1B). Compared to the rats in the CAD group, the hearts of the SEL (Figure 1C) and 2-APB (Figure 1D) groups showed fewer morphological alterations. Furthermore, compared to the SEL and 2-APB groups, the SEL+2-APB group (Figure 1F) rats’ hearts showed a considerable decrease in these morphological alterations.

### 3.2. Immunohistochemical Findings

Caspase-3 (Figure 1F) and TNF-α (Figure 1K) immunoreactivity were not observed in tissue sections of control groups. A significant increase in Casp-3 (Figure 1G) and TNF-α (Figure 1L) reactivity was observed in the CAD group compared to the control group. Casp-3 and TNF-α reactivity decreased in the SEL (Figure 1H,M) and 2-APB (Figure 1I,N) groups. In contrast, a significant decrease in Casp-3 (Figure 1J) and TNF-α (Figure 1O) reactivity was detected in the SEL+2-APB group. The intensity of antibody immunoreactivity in the groups is presented in Table 1.

### 3.3. TAS and TOS Levels in CAD-Induced Heart Damage

TAS (Figure 2A) and TOS (Figure 2B) levels were observed in heart tissue cell supernatants of all treatment groups. A significant decrease in TAS (Figure 2A) activity was observed in the CAD group compared to the control group (*p* < 0.05). An increase in TAS activity was detected in SEL and 2-APB groups. In contrast, a significant rise in TAS activity was detected in the SEL+2-APB group. A substantial increase in TOS (Figure 2B) activity was observed in the CAD group compared to the control group (*p* < 0.05). A decrease in TOS activity was detected in SEL and 2-APB groups. In contrast, a significant reduction in TOS activity was detected in the SEL+2-APB group.

### 3.4. MDA, GSH, GSH-Px and SOD Levels in CAD-Induced Heart Damage

**Figure 3:**
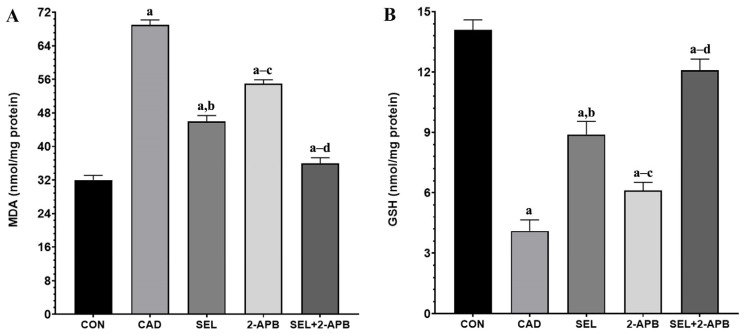
The impact of 2-Aminoethyl diphenylborinate (2-APB) and selenium (SEL) on malondialdehyde (MDA) (**A**) and glutathione (GSH) (**B**) values in cardiac damage caused by cadmium (CAD) (mean ± SD., and *n* = 8). [^a^ *p* < 0.05 comp. CON, ^b^ *p* < 0.05 comp. CAD, ^c^ *p* < 0.05 comp. SEL, ^d^ *p* < 0.05 comp. 2-APB groups, and *p* values were obtained according to one-way ANOVA test (post hoc Tukey) results].

**Figure 4:**
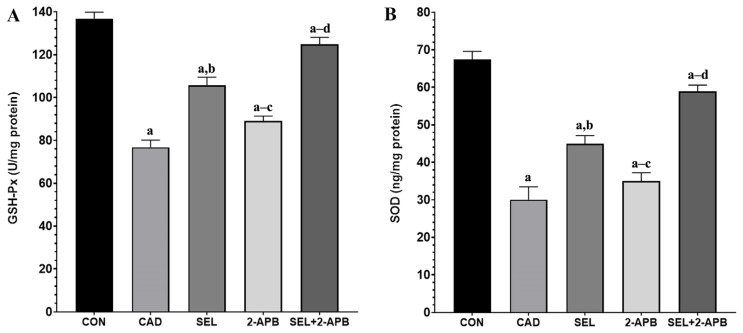
The impact of 2-Aminoethyl diphenylborinate (2-APB) and selenium (SEL) on glutathione peroxidase (GSH-Px) (**A**) and superoxide dismutase (SOD) (**B**) values in cardiac damage caused by cadmium (CAD) (mean ± SD., and *n* = 8). [^a^ *p* < 0.05 comp. CON, ^b^ *p* < 0.05 comp. CAD, ^c^ *p* < 0.05 comp. SEL, ^d^ *p* < 0.05 comp. 2-APB groups, and *p* values were obtained according to one-way ANOVA test (post hoc Tukey) results].

Malondialdehyde (MDA) (Figure 3A) and GSH (Figure 3B) levels were observed in heart tissue cell supernatants of all treatment groups. A significant increase in MDA (Figure 3A) activity was observed in the CAD group compared to the control group (*p* < 0.05). A decrease in MDA activity was detected in SEL and 2-APB groups. In contrast, a significant reduction in MDA activity was detected in the SEL+2-APB group. A significant decrease in GSH (Figure 3B), GSH-Px (Figure 4A), and SOD (Figure 4B) activity was observed in the CAD group compared to the control group (*p* < 0.05). An increase in GSH, GSH-Px, and SOD activity was detected in SEL and 2-APB groups. In contrast, a significant increase in GSH, GSH-Px, and SOD activity was detected in the SEL+2-APB group.

### 3.5. TRPM2 and PARP-1 Levels in CAD-Induced Heart Damage

**Figure 5:**
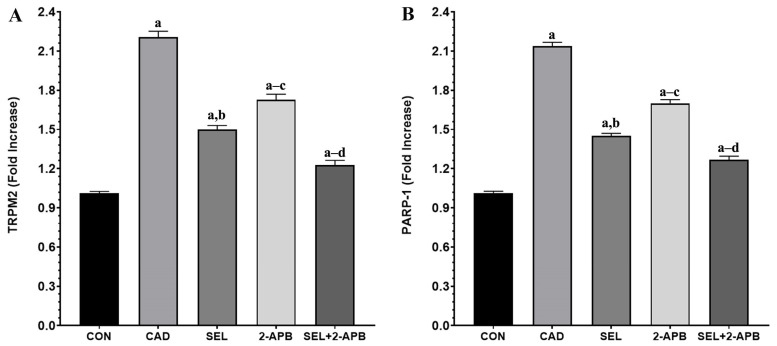
The impact of 2-Aminoethyl diphenylborinate (2-APB) and selenium (SEL) on transient receptor potential melastatin 2 (TRPM2) (**A**) and poly (ADP-ribose) polymerase 1 (PARP-1) (**B**) values in cardiac damage caused by cadmium (CAD) (mean ± SD., and *n* = 8). [^a^ *p* < 0.05 comp. CON, ^b^ *p* < 0.05 comp. CAD, ^c^ *p* < 0.05 comp. SEL, ^d^ *p* < 0.05 comp. 2-APB groups, and *p* values were obtained according to one-way ANOVA test (post hoc Tukey) results].

TRPM2 (Figure 5A) and poly (ADP-ribose) polymerase 1 (PARP-1) (Figure 5B) levels were observed in heart tissue cell supernatants of all treatment groups. A significant increase in TRPM2 and PARP-1 activity was observed in the CAD group compared to the control group (*p* < 0.05). A decrease in TRPM2 and PARP-1 activity was detected in SEL and 2-APB groups. In contrast, a significant reduction in TRPM2 and PARP-1 activity was detected in the SEL+2-APB group.

### 3.6. ROS and Casp-9 Levels in CAD-Induced Heart Damage

**Figure 6:**
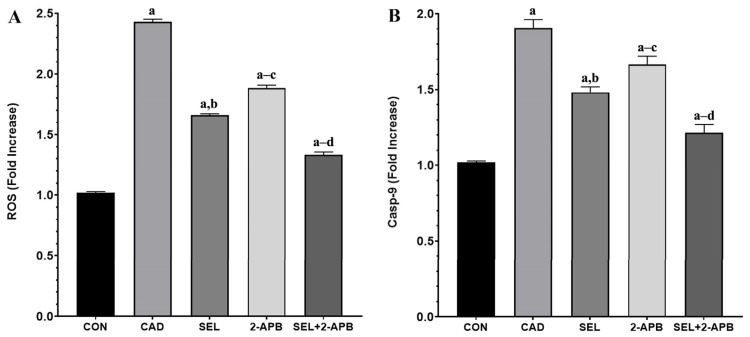
The impact of 2-Aminoethyl diphenylborinate (2-APB) and selenium (SEL) on reactive oxygen species (ROS) (**A**) and Casp-9 (**B**) values in cardiac damage caused by cadmium (CAD) (mean ± SD., and *n* = 8). [^a^ *p* < 0.05 comp. CON, ^b^ *p* < 0.05 comp. CAD, ^c^ *p* < 0.05 comp. SEL, ^d^ *p* < 0.05 comp. 2-APB groups, and *p* values were obtained according to one-way ANOVA test (post hoc Tukey) results].

ROS (Figure 6A) and Casp-9 (Figure 6B) levels were observed in heart tissue cell supernatants of all treatment groups. A significant increase in ROS and Casp-9 levels was observed in the CAD group compared to the control group (*p* < 0.05). A decrease in ROS and Casp-9 levels was detected in SEL and 2-APB groups. In contrast, a significant reduction in ROS and Casp-9 levels was detected in the SEL+2-APB group.

## 4. Discussion

Heavy metal poisoning and its effects are becoming more prevalent in the contemporary global environment. According to several earlier studies, CAD is the most hazardous heavy metal. All living things, including humans, are harmed by CAD’s generation of free radicals and cellular OS [9,30]. It is regarded as a public health problem since it affects nearly all essential organs. One of the major negative health impacts of CAD exposure is cardiotoxicity, which has been shown to accumulate in both human and animal hearts. It has been recognised that OS and inflammation play a role in mediating myocardial damage and apoptosis brought on by exposure to CAD [4,13]. Myocardial infarction, cardiomyopathy, peripheral artery disease, stroke, hypertension, arteriosclerosis, and heart failure are among the cardiovascular conditions that have been linked to CAD, according to a number of earlier studies [8,31].

SEL is a trace mineral that is essential for maintaining heart health. It is an essential part of GSH-Px, which controls the body’s redox process by affecting GSH-Px. According to studies, keeping SEL levels in check can lower the risk of cardiovascular illnesses [32]. Given SEL’s health benefits, this study examined how it affected the inflammatory response, lipid peroxidation, and ROS caused by CAD in rats’ hearts. SEL and 2-APB reduced myocardial damage by inhibiting OS, the PARP-1/ADPR/TRPM2 pathway, and the following apoptosis and cell death. We assessed the impact of SEL treatment on the TRPM2-channel-mediated signalling pathway in the rat myocardial cells, both alone and in combination with 2-APB. Our results showed that CAD encouraged excessive Ca^2+^ influx, which led to OS, elevated ROS, and an ROS-induced increased expression of TRPM2 (the TRPM2 channel’s Nudix box domain is sensitive to ROS). The increase in cytosolic Ca^2+^ led to decreased cytosolic GSH, GSH-Px, and SOD levels and increased cytoplasmic ROS production. Moreover, elevated Ca^2+^ influx led to apoptosis and cell death in the rats’ hearts by activating caspase pathways, such as Casp-3 and Casp-9. As shown in the graphical abstract, we found that SEL and 2-APB act synergistically on the heart of rats through the molecular pathways.

Lipid peroxidation results from oxidative dysfunction and free radicals produced by indiscriminate exposure to CAD. This is linked to a change in the antioxidant defence system, which is followed by oxidative damage to proteins, DNA, and lipids. Our model shows a considerable rise in MDA levels, the most accurate indicator of OS and lipid peroxidation [33,34]. Antioxidant enzymes are thought to be the first line of defence against OS to preserve cellular integrity, protect cell membranes, and stop the onset of various degenerative illnesses. Due to interaction with the excessively generated free radicals, the levels of enzymatic antioxidant enzymes, particularly GSH-Px (such as SOD, catalase), and the non-enzymatic antioxidant GSH, drop under OS [35,36].

Increased inflammation and a disrupted antioxidant/oxidant balance will impair contractile function, making it more difficult for the heart to pump blood. Serious conditions like hypotension, shock, and even heart failure could arise as a result [37]. All of these conditions have one thing in common: the heart’s increased workload cannot be made up for. As a result, tissues experience ischemia and OS due to reduced oxygenation brought on by impeded circulation. Tissue damage results from an inadequate antioxidant response brought on by elevated ROS from OS [38]. According to the analysis results made in light of this information, MDA and TOS levels increased in the CAD group due to CAD toxicity, while TAS, GSH, GSH-Px, and SOD levels decreased (compared to CON, *p* < 0.05). Furthermore, SEL may also have protective benefits in physiological settings that are not related to tissue injury, as indicated by the SEL group’s lowest TOS and greatest TAS, GSH, GSH-Px, and SOD levels.

The findings of the study showed that CAD greatly reduced the levels and activities of TAS, GSH, GSH-Px, and SOD in the cardiac tissues treated by CAD, but that TOS and MDA levels were much higher in the same cardiac tissues. According to this study, the hearts of rats given CAD may have an excessive amount of lipid peroxidation and increased protein carbonyl formation due to oxidative modifications of proteins and lipids in the heart tissue brought on by a decline in antioxidant defence and excessive free radical generation. Because of its peroxidative properties, CAD causes OS that breaks down lipids and other macromolecules. It is well known that CAD action primarily targets cellular membranes, resulting in lipid peroxidation [39]. It is a serious procedure that damages a number of tissues and organs, most notably the kidneys and heart. These findings are consistent with other reports [40,41]. Since CAD-treated rats showed changes in the enzyme activity that constitute the first line of defence against O_2_ and H_2_O_2_, OS is linked to the activation of lipid peroxidation. Our findings showed that CAD-treated rats had greater levels of oxidants (TOS), as indicated by increased levels of MDA in heart tissue. This could lead to the death of cardiac tissue in rats subjected to CAD. The results of the current study indicate that SEL has a protective effect since a co-treatment of SEL with CAD restores the changed levels of all examined antioxidant enzymes in rats. Furthermore, because SEL can scavenge ROS, it was able to preserve the non-enzymatic antioxidant state and prevent lipid peroxidation in heart tissue. In the present study, we included 2-APB to further investigate the role of TRPM2 channels in CAD-induced cardiotoxicity. As a known TRPM2 antagonist [24,42], 2-APB allowed us to assess the functional relevance of TRPM2 activation in this pathological process. Our findings showed that treatment with 2-APB significantly attenuated oxidative stress markers and cellular damage, supporting the involvement of TRPM2 in cadmium-induced cardiotoxicity. These results strengthen the hypothesis that TRPM2 channels play a mechanistic role in mediating oxidative stress and calcium dysregulation in cardiac tissue, and that the pharmacological inhibition of TRPM2 may represent a potential therapeutic strategy. In addition, the co-administration of 2-APB, a TRPM2 channel antagonist, and SEL created a synergistic effect against the cellular toxicity caused by CAD, making the treatment more effective.

Caspase family proteins are measured to assess the stimulation of the apoptotic cascade that follows the occurrence of oxidative damage and inflammation. When free radicals and other pro-inflammatory agents like TNF-α and NFκB are stimulated, an imbalance between pro-apoptotic and anti-apoptotic factors results in programmed cell death. The release of multiple caspases, including Casp-3, the most significant marker of apoptosis, follows this. Furthermore, both intrinsic and extrinsic routes depend on Casp-3 [43,44]. When a specific ligand binds to the death receptor, Caspase-8 is activated, which either directly triggers apoptosis by activating executioner caspases or triggers the intrinsic apoptotic pathway as a result. Numerous cellular stressors can trigger the intrinsic or mitochondrial apoptosis pathway, which causes cytochrome c to be released from the mitochondria and the apoptosome, which is made up of APAF1, cytochrome c, ATP, and Casp-9, to form. This activation of Casp-9 follows. Then, by cleaving and triggering executioner caspases, active Casp-9 starts apoptosis [45,46]. In line with earlier research, we assessed the apoptotic markers Casp-3 and TNF-α immunoexpression in the CAD group. The results showed a considerable increase in these markers’ expression, indicating the occurrence of apoptosis brought on by CAD administration [47]. The CAD treatment caused significant increases in Casp-3/9 and TNF-α reactivity, whereas a substantial decrease in Casp-3/9 and TNF-α reactivity was detected after separate administrations of SEL and 2-APB treatments. On the other hand, in the SEL+2-APB group, the co-administration of SEL and 2-APB treatments made the treatment even more effective.

Biochemical and histopathological investigations revealed heart injury in CAD-challenged rats. After CAD exposure, membrane integrity was disrupted, and leakage of harmful enzymes out of the cardiomyocyte was caused. Histologic examination findings revealed that the morphologic changes in the hearts of CAD group rats were characterised by marked necrosis and the degeneration of myocytes and congestion of vessels. In addition, typical inflammatory infiltration features were present [48]. SEL and 2-APB prevented cell damage and successfully reduced the detrimental effects of CAD on the heart. These results demonstrated the cardioprotective effect of SEL and corroborated earlier research that demonstrated its protective function against myocardial injury caused by lipopolysaccharide [49], as well as T-2 toxin [32] and other factors. The impact of SEL on these pathological processes was examined since redox imbalance and inflammation are implicated in the harmful effects of CAD, including cardiotoxicity. Free radicals are not directly produced by redox reactions when CAD is in the +2-oxidation state. On the other hand, CAD indirectly produces superoxide and hydroxyl radicals, H_2_O_2_ and NO [50]. Through Fenton-type mechanisms, CAD increases the formation of ROS by promoting the release of free iron [51]. Consequently, cardiomyocytes [13] and other cells, including hepatocytes [50], have higher levels of cellular ROS after being exposed to CAD. By oxidising lipids, proteins, and DNA, excess ROS can harm cellular macromolecules, leading to lipid peroxidation, a reduction in antioxidants, and cell damage. Furthermore, ROS trigger additional ROS formation and cell death by promoting several signalling pathways, such as PARP-1/ADPR/TRPM2, and subsequently inflammatory mediators [22,24]. It has already been established from several investigations that cell injury increases the expression of the TRPM2 channel. According to our study’s biochemical findings, the application of CAD raised the expression of TRPM2 channels in heart tissue. In our study, both TRPM2 channel expression and PARP-1-mediated TRPM2 channel expression levels were increased in cardiac tissue, consistent with the literature, suggesting an association with ROS levels [22,23]. The enzymatic activity of PARP-1 produces ADPR intracellularly in the cell nucleus. An indication of cell nucleus damage is the rising PARP-1. Furthermore, the elevated intracellular ADPR is shown by PARP-1. TRPM2 channel activity is also increased by intracellular increases in ADPR [21,24]. We found that pro-inflammatory cytokines rose and antioxidant levels dropped in the rat heart tissues following CAD treatment. We found that the levels of ROS in cardiac tissue increased concurrently with the rise in pro-inflammatory cytokines. In heart tissue samples, we also noticed elevated PARP-1 levels. We found that TRPM2 channel expression increased in tandem with the rise in pro-inflammatory cytokines, ROS, and PARP-1. We found that the administration of CAD in conjunction with SEL and/or 2-APB therapy reduced the levels of ROS, PARP-1, and TRPM2 channels in cardiac tissue.

In rats with CAD, cardiac OS was indicated by increased MDA, TNF-α, TOS, and ROS, and decreased GSH, GSH-Px, SOD, and TAS. Elevated ROS oxidize DNA and acti-vate the PARP-1/ADPR/TRPM2 pathway [22]. Antioxidant enzymes include preventing ROS-mediated oxidative damage, neutralising ROS, and preserving the integrity of cell membranes. Consequently, diminished levels of these defences make the cells vulnerable to death. In vivo investigations showed a reduction in antioxidants after exposure to CAD [20,26], and CAD has been shown to increase ROS and impair cardiomyocyte cell viability [13]. In light of the explanations, CAD treatment caused significant increases in ROS, PARP-1, and TRPM2 levels (Compared to CON, *p* < 0.05). In contrast, a significant decrease in ROS, PARP-1, and TRPM2 levels was detected after separate administration of SEL and 2-APB treatments. On the other hand, in the SEL+2-APB group, the co-administration of SEL and 2-APB treatments made the treatment even more effective. Therefore, in this study, we demonstrated for the first time that one of the molecular pathways for the protective effects of SEL and 2-APB against CAD-induced cardiac injury may be the ROS-induced PARP-1/ADPR/TRPM2 pathway in rat cardiac cells.

The doses used in this study were selected based on previous toxicological models to elucidate the mechanistic pathways of CAD-induced cardiotoxicity and the protective role of SEL. Our findings suggest that SEL may mitigate oxidative stress and TRPM2-mediated injury via modulation of the ROS/PARP-1/TRPM2 signalling axis. These insights may guide future therapeutic strategies targeting oxidative stress-related cardiac damage. However, it is important to note that SEL has a narrow therapeutic window, and higher doses may lead to toxic effects. Therefore, caution should be exercised when considering SEL supplementation, especially in clinical contexts. Future studies should explore the effects of chronic, low-dose SEL administration and assess its safety profile through pharmacokinetic and toxicological analyses. To improve translational relevance, upcoming research should also incorporate chronic exposure models using environmentally relevant doses of CAD and SEL. This will provide a more accurate foundation for designing dose-adjusted and safe human clinical trials to mitigate heavy metal-induced cardiotoxicity.

## 5. Conclusions

This study shows that CAD can cause apoptosis in rats by raising levels of pro-inflammatory cytokines, ROS, PARP-1, and TRPM2 channels, which will harm the heart’s architecture and function. Apart from these apoptotic indicators, other results from this investigation demonstrated that CAD-induced cardiac damage was mediated by pro-inflammatory cytokines, ROS, PARP-1, and TRPM2. However, by blocking these parameters, the combination of SEL and/or 2-APB reduced cardiac damage. However, it was shown that TRPM2 channel blocking with 2-APB and SEL therapy in conjunction with CAD may protect against the cardiotoxic consequences of CAD. Apoptosis markers, antioxidant parameters, and parameters related to TRPM2 channel activation must be examined at the molecular level to gain a better understanding of how CAD affects cardiotoxicity and to look into the potential protective effects of SEL treatment and TRPM2 channel blockade in conjunction with CAD.

## Figures and Tables

**Table 1:** The intensity of the Caspase-3 and TNF-α immunoreactivity in the rat heart tissues.

Groups					
Antibodies	CON	CAD	SEL	2-APB	SEL+2-APB
**Casp-3**	**-**	**+++**	**++**	**+**	**+**
**TNF-α**	**-**	**+++**	**+**	**++**	**+**

Negative (**-**), mild (**+**), moderate (**++**), and intense (**+++**).

## Data Availability

The data that support the findings of this study are available from the corresponding author upon reasonable request.
